# Founding Editorial – Forensics and TheScientificWorld

**DOI:** 10.1100/tsw.2001.299

**Published:** 2001-10-30

**Authors:** Walter Rowe

**Affiliations:** Department of Forensic Sciences, The George Washington University, 2121 Eye Street N.W., Washington, D.C. 20052, USA

**Keywords:** forensics, forensic sciences, DNA analysis, DNA profiling, crime scene processing, crime laboratories, quality control, quality assurance

## Abstract

At the beginning of a new millennium it seems a good idea to stop for a moment and take stock of the current state of forensic science. As a field of scientific research and scientific application, forensic science is a little more than a century old. Forensic science may be said to have begun in 1887 with the simultaneous publication of A. Conan Doyle’s A Study in Scarlet and Hans Gross’s Handbuch für Untersuchungsrichter. Conan Doyle’s novel introduced to the world the character of Sherlock Holmes, whose literary career would popularize the use of physical evidence in criminal investigations. Gross’s manual for examining magistrates suggests ways in which the expertise of chemists, biologists, geologists, and other natural scientists could contribute to investigations. Gross’s book was translated into a number of languages and went through various updated editions during the course of the century. The intervening century saw the development and application of fingerprinting, firearm and tool mark identification, forensic chemistry, forensic biology, forensic toxicology, forensic odontology, forensic pathology, and forensic engineering. Increasingly, the judicial systems of the industrial nations of the world have come to rely upon the expertise of scientists in a variety of disciplines. In most advanced countries, virtually all criminal prosecutions now involve the presentation of scientific testimony. This has had the beneficial effect of diminishing the reliance of courts on eyewitness testimony and defendant confessions.

The most significant advance in forensic science in the last two decades has been the development of DNA profiling. The impact of DNA profiling on the criminal justice system has been profound. When DNA profiling first began to be applied in criminal prosecutions it was immediately apparent that this was an extremely powerful tool for criminal identification; while in most circumstances conventional genetic marker analysis of blood and other body fluids might narrow down the possible source of a blood or semen stain to 1% of the general population, DNA profiling using restriction fragment length polymorphisms (RFLP) could easily reduce the probability of a random match to a member of the general population to 1 in 100 million. With the advent of polymerase chain reaction (PCR)-based DNA profiling methods, forensic scientists could at last extract significant information from very small, very dilute or very degraded body fluid stains. Although the gene loci used in PCR-based DNA profiling are individually less informative than the RFLP loci, PCR permits use of a large number of loci so that the source of the DNA in a blood or body fluid stain can be definitively determined. PCR-based mitochondrial

DNA sequencing has also found important applications in the identification of human remains and in the comparison of human and animal hair. The discriminating power of DNA profiling has made it feasible to establish databases of convicted felon DNA profiles which can be searched when DNA is recovered at the scene of a crime. The nationwide database has led to the solution of a number of unknown subject sexual assault cases; it has also allowed the linking of unsolved cases in different jurisdictions. New DNA technology has also permitted law enforcement agencies to re-open ‘cold cases’ — old, unsolved cases. Unknown murder victims have been identified decades after their bodies were found and cases have also been successfully developed against suspects who believed that they had literally gotten away with murder.

The advent of DNA profiling has also emphasized to the judicial system and to the public that forensic science can be and ought to be impartial and unbiased, and that it can aid the accused and the unjustly convicted. The Federal Bureau of Investigation has found that in nearly one third of the DNA cases its laboratory handles the principal police suspect is excluded as the source of the DNA from the crime scene or victim. DNA testing has also exonerated a number of unjustly convicted defendants. The Innocence Project, established by prominent defense attorney Barry Scheck, has successfully used post-conviction DNA testing to exonerate a number of defendants who had been convicted of the most serious crimes, including capital murder. In fact, post-conviction DNA testing has raised serious questions about the imposition of the death penalty; for example, the governor of Illinois imposed a moratorium on executions when post-conviction DNA testing caused Illinois to release more condemned men than it executed. Some states in the U.S. have passed laws granting defendants access to evidence for post-conviction testing. Post-conviction DNA testing has also starkly exposed the bias, dishonesty, and incompetence of some police investigators, prosecutors, and forensic scientists. It is a case of good forensic science driving out the bad.

The development of DNA profiling has had indirect effects on other forensic science disciplines. For years forensic scientists in many disciplines have operated without commonly agreed upon methods of analysis or standards of interpretation. Although ASTM committee E30 on forensic science has existed since the 1970s it had not promulgated many consensus standards until the 1990s. Committee E30 has now published voluntary consensus standards in forensic biology, forensic chemistry, and document examination. The pressure exerted by courts for standard DNA analysis methods led the FBI to establish the Technical Working Group for DNA Analysis Methods (TWGDAM), a working group composed of both forensic science practitioners and academics. TWGDAM developed standards for the education and training of DNA analysts and DNA supervisors and standards for the analysis of biological evidence using RFLP and PCR methods. Similar technical working groups (TWGs) and scientific working groups (SWGs) have been established in many other forensic science disciplines. These groups are beginning to promulgate similar standards for education, analytical methods, and interpretation for their disciplines.

DNA testing as well as high-profile criminal cases such as the O.J. Simpson Case have placed a new emphasis on the training of those charged with the responsibility for crime scene evidence collection. The O.J. Simpson Case demonstrated that excellent forensic science (especially the DNA analysis performed by Cellmark Diagnostics and the footwear impression examination performed by the FBI) cannot prevail in the face of questionable evidence collection procedures. The National Institute of Justice has issued detailed guidelines for death investigations, general crime scene processing and arson investigation. Many law enforcement agencies have recognized that crime scene processing, is so specialized an endeavor that it should be the exclusive responsibility of specially trained police officers or crime scene technicians. Professional organizations have also begun to develop certification programs for crime scene processors.

Another example of what may be referred to as ‘collateral damage’ produced by the introduction of DNA profiling is the revived interest in the formalization of quality assurance policies and quality control procedures in forensic science laboratories. Increasing numbers of forensic science laboratories in the U.S. are becoming accredited through the American Society of Crime Laboratory

Directors–Laboratory Accreditation Board (ASCLD-LAB). The Council of Forensic Educators may also begin to accredit forensic science education programs in the near future. Many laboratory examiners in the U.S. are also having their expertise in their disciplines certified by the American Board of Criminalistics (ABC). The laboratory accreditation program of ASCLD and the forensic examiner certification program of ABC emphasize continued proficiency testing to insure the quality of laboratory case work. Many forensic laboratories now routinely subject their examiners to internal and external proficiency tests. A number of professional organizations and commercial firms have created proficiency test kits for various forensic science disciplines.

The practice of forensic science has also been influenced by a changing attitude toward science and scientists among judges. For most of the 20$^{th}$ century the guiding principle for the admission of scientific evidence in U.S. courts was the ‘general acceptance’ criterion enunciated by the DC Court of Appeals in *Frye v. United States* in 1923. According to the Frye Rule the results of a scientific test would be admissible in court if the test enjoyed general acceptance in the scientific field to which it belonged. In 1993 the U.S. Supreme Court handed down its decision in the case of *Daubert et al. v. Merrell Dow Pharmaceuticals*. This decision overturned the Frye Rule and replaced it with a more searching and skeptical inquiry into the basis for the testimony of a scientific expert. Questions to be asked of novel scientific testimony included the following: Has this technique or theory been tested? Can it be tested? Has it been subjected to peer review and published? What is its error rate? Do appropriate standards exist for the performance of tests? Has it gained general acceptance in the scientific field to which it belongs? In *Kumho Tire Company, Ltd. et al. v. Patrick Carmichael etc. et al*. the U.S. Supreme Court later extended these guidelines to fields such as engineering, where experience and ‘rules of thumb’ had previously been deemed adequate bases for expert opinions. Although the Daubert and Kumho decisions are strictly speaking authoritative only in the Federal courts of the U.S. their impact has been far reaching. DNA profiling was able to meet the standards of Daubert easily. Other widely used (and hitherto widely accepted) forensic science tests, however, have been subjected to challenges under Daubert, including fingerprint identification and handwriting comparisons. Such Daubert challenges emphasize the need for forensic science to re-assess the scientific bases of much that forensic examiners do.

The *Forensics* domain of *TheScientificWorld* will provide a forum for review articles. In its early issues there will appear a series of invited review articles from leading experts in a range of forensic science disciplines. In due course other reviews will be added until the full range of forensic science is represented. A unique feature of *Forensics* will be the frequent updating of these review articles as the forensic science field continues to evolve. From time to time *Forensics* will also publish commentaries on important issues in forensic science. Another important goal of *Forensics* will be to provide a venue for research articles. The Editors are committed to maintaining the highest standards for scientific peer-review, a rapid review and publication process (which is particularly significant for such a rapidly evolving field), a flexible publishing platform which will support features not available in standard journals (e.g., video and audio clips of experiments or reconstructions, high-resolution graphics or downloadable data and software files) and access to a global audience. We look forward to creating a new mode for forensic science communication.

**Walter F. Rowe**
Principal Editor, *Forensics* Domain

## EDITORIAL BOARD for

## FORENSICS

**A Domain of *TheScientificWorld***

### PRINCIPAL EDITOR

**Prof. Walter F. Rowe**
Department of Forensics, George Washington University, DC, U.S.

### ASSOCIATE EDITORS

**Dr. Richard Saferstein,** Forensics Science Consultant, Mt. Laurel, NJ, U.S.

**Dr. Edward M. Robinson,** Department of Forensic Sciences, George Washington University, DC, U.S.

### EDITORIAL BOARD MEMBERS

**Dr. E. Roland Menzel,** Center for Forensic Studies, Texas Technical University, TX, U.S.

**Dr. Arie Zeichner,** Israel Police National Headquarters, Jerusalem, Israel
